# Lactation transcriptomics in the Australian marsupial, *Macropus eugenii*: transcript sequencing and quantification

**DOI:** 10.1186/1471-2164-8-417

**Published:** 2007-11-13

**Authors:** Christophe M Lefèvre, Matthew R Digby, Jane C Whitley, Yvan Strahm, Kevin R Nicholas

**Affiliations:** 1 CRC for Innovative Dairy Products, Department of Zoology, University of Melbourne, VIC, 3010, Australia; 2 Victorian Bioinformatics Consortium, Monash University, Clayton VIC 3080; 3 Department of Primary Industries, 475 Mickleham Rd, Attwood, VIC 3045, Australia

## Abstract

**Background:**

Lactation is an important aspect of mammalian biology and, amongst mammals, marsupials show one of the most complex lactation cycles. Marsupials, such as the tammar wallaby (*Macropus eugenii*) give birth to a relatively immature newborn and progressive changes in milk composition and milk production regulate early stage development of the young.

**Results:**

In order to investigate gene expression in the marsupial mammary gland during lactation, a comprehensive set of cDNA libraries was derived from lactating tissues throughout the lactation cycle of the tammar wallaby. A total of 14,837 express sequence tags were produced by cDNA sequencing. Sequence analysis and sequence assembly were used to construct a comprehensive catalogue of mammary transcripts.

Sequence data from pregnant and early or late lactating specific cDNA libraries and, data from early or late lactation massively parallel sequencing strategies were combined to analyse the variation of milk protein gene expression during the lactation cycle.

**Conclusion:**

Results show a steady increase in expression of genes coding for secreted protein during the lactation cycle that is associated with high proportion of transcripts coding for milk proteins. In addition, genes involved in immune function, translation and energy or anabolic metabolism are expressed across the lactation cycle. A number of potential new milk proteins or mammary gland remodelling markers, including noncoding RNAs have been identified.

## Background

After a short gestation period, marsupials give birth to a relatively immature newborn that is totally dependant on milk for growth and development during a relatively long lactation period. Important changes occur during the lactation cycle of marsupials in terms of mammary gland development, milk production, milk composition as well as development or behaviour of the young [1]. By contrast, eutherian mammals have evolved with a larger investment in the development of the young during gestation [2] and, apart from the initial colostrum during the immediate postpartum period, produce milk of a relatively constant composition [3]. Marsupial milk provides essential nutrients and putative growth factors for the development of the young and crossfostering experiments have shown that milk controls post-natal development [4,5].

Endocrine and others factors, potentially intrinsic to the mammary gland, are likely to control milk secretion [6] and marsupial milk contains autocrine/paracrine regulators of the mammary gland [7,8]. In special circumstances macropod marsupials such as the tammar wallaby *Macropus eugenii *and red kangaroo *Macropus rufus *may present asynchronous concurrent lactation, feeding concurrently two young of different ages with milk of different compositions from adjacent mammary glands; a new born pouch young and an few months older animal [9,10]. However, the molecular control mechanisms of marsupial milk composition are not fully known.

The tammar wallaby (*Macropus eugenii*) is an annual breeder characterised by a short pregnancy lasting 26 days followed by an extended lactation period of about a 300 days. The lactation cycle is divided into 3 phases of approximately 100 days each based on the sucking pattern of the young (permanently attached to the teat, permanently in the pouch and intermittently sucking, in and out of the pouch) and milk composition. Shortly after birth, the single young weighing only 400 mg crawls into the pouch and attaches to one of four teats, each associated with a separate mammary gland. The chosen teat will provide all the milk during the entire period of lactation with massive growth of the associated glandular tissue while the other three glands do not generally participate in any milk production.

Changes in expression levels of milk protein genes have been described for a number of milk proteins in several marsupial species. In particular, lactation stage specific genes, such as early lactation protein (ELP), mid-late whey acidic protein (WAP) and late lactation proteins (LLP-A and LLP-B) have been characterised in the tammar and other species [11-18]. Interestingly, with the exception of WAP which is also found in milk of many eutherians [19] but not in humans, goat and ewe [20], all of these marsupial phase specific milk proteins as well as another ubiquitous marsupial milk protein, trichosurin [21] have not been found in eutherian milk. We now report expression of these and other new potential milk protein genes quantified by sampling the mammary transcriptome at specific stages of the tammar lactation cycle.

## Results and discussion

### Library construction, EST sequencing and annotation

A comprehensive set of cDNA libraries was derived from mammary tissue collected throughout the lactation cycle of the tammar: 9 stage specific libraries from pregnant day 23 (n = 4) or lactating animals at day 130 (n = 4) and day 260 (n = 1), 2 libraries from day 130 of lactation subtracted for major milk genes (alpha-lactalbumin, betalactoglobulin, alpha-casein, beta-casein and kappa-casein), 2 libraries from the nonlactating glands (no young attached to the teat) of day 4 lactating animals and, a normalized library from a mix of mammary tissues (day 55,87,130,180,220,260 and 5 days involution after 45 days of lactation). A total of 14,837 expressed sequence tags (EST) were characterized by DNA sequencing (Table 1), including 8,027 from the normalised [GenBank:EX195538 .. EX203564] and 6,810 from lactation phase specific libraries [GenBank:EX203644 .. EX210452]. BLAST searches [22] (E-value < 1.e-10) resulted in hit rates of 50% against the non-redundant protein database, 60% against the genome of the American marsupial opossum *Monodelphis domestica *and, 85% against the sequence archive of the *Macropus eugenii *genome trace archive at NCBI. Furthermore, poorly annotated 3'ESTs often matched 3'non-coding regions of annotated genes from the opossum Ensembl genome server [23] with lower E-values.

**Table 1:** cDNA library data set

Library	ESTs	lactation stage
01	227	day130L
02	81	day130L
03	121	day130L
04	218	day130L
11	369	day130L-substracted
12	443	day130L-substracted
13	1887	day130L
14	76	day260L
15	162	day23p
16	268	day23p
17	1939	day23p
18	458	day4L unsucked
19	559	day4L unsucked
20	8027	normalised
Total	14836	

### Sequence assembly and gene clustering

EST clustering and sequence assembly were used to construct a comprehensive catalogue of transcripts. The CAP3 sequence assembly program [24] produced 2,007 contigs and 5,478 singletons. Furthermore, BLAST was used to detect groups of high similarity within contig sequences. A score cut off value of 170 resulted in the grouping of 160 contigs into 55 super-contigs. Additional validation was performed by inspection of multiple alignments to separate gene family clusters (eg: cathelicidins), identify splicing variants (eg. alpha-casein) and check for artefacts such as chimeras, incorrect vector clipping error, simple sequences or sequence rearrangements. The final edited gene index contains 1929 clusters from 1848 original CAP3 contigs and 45 validated supercontigs. During this analysis 2 putative splice variants were identified in alpha-casein resulting in the in frame deletion of 8 (aa 82–89) and 14 (aa 125–138) amino acid fragments corresponding to short exons in other species. In addition the alpha-casein C terminal sequence has been improved and found to be much more similar to the possum (*Trichosurus vulpecula*) sequence than previously reported [25]. Automatic contig sequence annotation using BLAST searches (E-values < e-10) resulted in similar hit rate of about 60% against a number of databases; the non redundant protein database, the set of GenScan protein predictions from the opossum genome, the complete opossum genome sequence and 49% with the Ensembl cDNA predictions from the human genome. However, 94% of contig sequences could be retrieved from the partial genome sequence archive of the tammar wallaby.

### Multi species comparison of mammary transcript sequences

In order to investigate the level of similarity between the tammar wallaby and other vertebrate cDNAs, BLASTN was used to identify best hits in Unigene sequence data sets (dog, rat, mouse, cow) or ENSEMBL genome cDNA predictions from a number of species (tenrec, man, chicken, armadillo, zebrafish, opossum and platypus).

**Figure 1:**
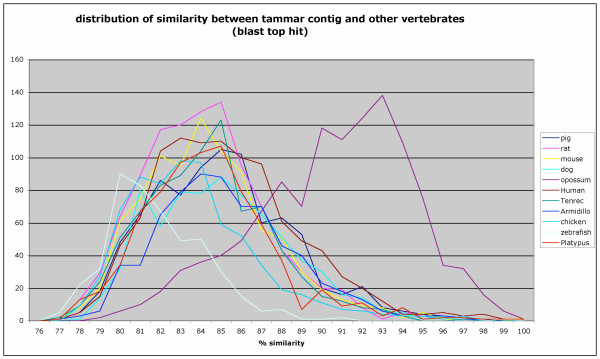
**Distribution of sequence identity between tammar contig and other vertebrates**. The BLASTn algorithm was used to identify best matches (top hits) between tammar contig sequences and Unigene sequence data sets (dog, rat, mouse, cow) or cDNA sequences retrieved from the ensembl annotation of a number of species (tenrec, man, chicken, armadillo, zebrafish, opossum and platypus). Percentage sequence similarity of top hit BLAST alignments were retrieved from the BLAST output, filtering for alignments longer than 100 nucleotides with score greater than 170.

Sequence identity distributions of best score hit alignments are represented in Figure 1. It can be seen that the distribution of similarity between the tammar and the other marsupial, the opossum *Monodelpis domestica *is much higher (average similarity 90.5%) than the similarity between the tammar and any other mammal including eutherian species (84.2–85.3%), the monotreme platypus (84.4%) and more distantly related vertebrates such as the chicken (83.6%) and the zebrafish (81.9%). In addition, the proportion of tammar contigs mapped across species varies from 23% in zebrafish or 35% in chicken to 59% with the marsupial opossum, while 49% can be mapped to human mRNA sequences and 38% in the platypus. These results have to be considered carefully due to the heuristic nature of alignment selection by the BLAST algorithm. However, since only relatively long alignments were retained, this provided an estimate of similarity distributions between tammar mammary transcripts and the genomes of other species and is compatible with other evolutionary evidences [26]. It also suggests that up to 10% of the mammary transcriptome may be marsupial specific (contigs found in the opossum not found in human) and up to an additional 15% therian or mammalian specific (gene found in human not found in chicken). In this analysis the platypus results are disregarded because the platypus gene set based on automatic gene prediction is not complete.

### Estimation of gene expression from lactation stage specific libraries

**Figure 2:**
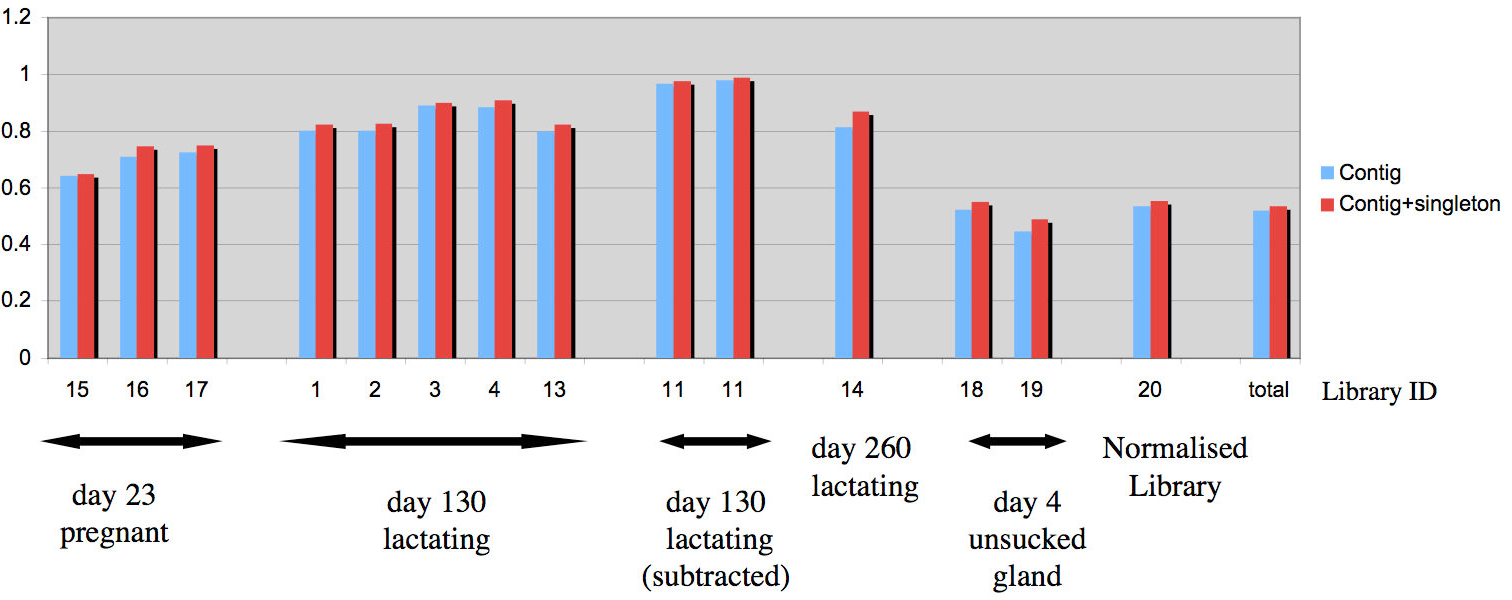
**Proportion of EST sequences assigned to contigs in each library**. The proportion of ESTs assigned to contigs in each library is shown when ignoring (blue) or including (red) an additional 360 singletons with high similarity to contig sequences (BLAST score > 170). Inclusion of such singletons does not have a significant effect because they mainly represent chimeras associated with highly expressed milk transcripts. Interestingly, in all libraries from a milk-producing gland, more than 80% of ESTs are asssigned to contigs irrespective of lactation phase (day 130 or day 260) or sampling depth (76–1,887 ESTs). Thus a large proportion of transcripts from lactating tissue are covered by multiple EST counts in the data.

**Figure 3:**
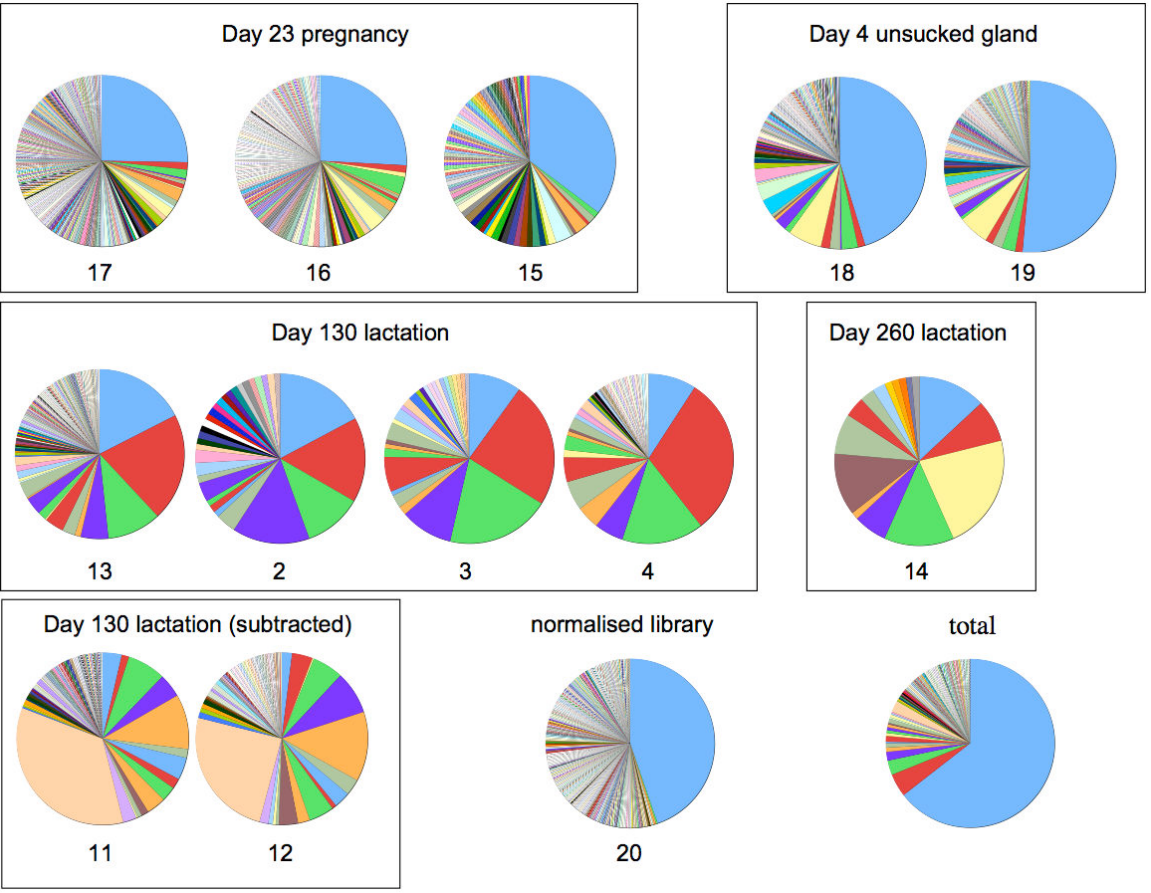
**EST library composition**. Relative abundance of transcript was calculated by estimating the frequency of each transcript from the number of overlapping ESTs mapping in each library (normalized to parts per 10000). cDNA Libraries are grouped by lactation stage and library number are indicated below each pie chart. Total represents the pool of all ESTs from all libraries. Colour coding of genes is presented in Figure 4.

Lactation stage specific mammary transcript frequencies were evaluated from the distribution of ESTs within each stage specific library. This approach provides an estimation of the relative abundance of major gene transcripts in the mammary gland which may be sampled multiple times in any cDNA library. Figure 2 shows the proportion of ESTs assigned to contigs in each library when ignoring or including an additional 360 singletons with high similarity to contig sequences (BLAST score > 170). Inclusion of singletons does not have a significant effect because they mainly represent chimeras associated with highly expressed transcripts. Interestingly, in all libraries from a milk-producing gland, more than 80% of ESTs are assigned to contigs irrespective of lactation phase (day 130 or day 260) or sampling depth (76–1887 ESTs). Remarkably, the high coverage obtained with day 130 subtracted libraries is close to 100%, suggesting that a large proportion of the singletons at day 130 in other day 130 libraries are derived from cloning and sequencing artefacts induced, for the most part, by the high abundance of otherwise subtracted casein and betalactoglobulin milk protein transcripts. The coverage in glands from pregnant animals was also consistently lower at 60 to 78%, and even much lower at 40–50 % in unsucked glands as well as in the combined and normalised libraries. These results show that the sequence data capture a large proportion of the transcript population in lactating glands and suggests a shift of transcriptional complexity during the lactation cycle. The transcript composition of each cDNA library is represented in Figure 3.

Generally, it can be seen that the composition is similar in libraries from related lactation stages or sample preparations despite differences in the sampling depth. The second observation is that, as previously noted, the proportion of singletons is highest in samples from un-sucked and pregnant glands (~50% and 25–30% respectively). In contrast, all lactating samples contain less than 20% singletons. Conversely, any major transcript in pregnant and un-sucked gland samples does not represent more than a few percent while the major milk protein gene may represent more than 25% of all transcripts in un-substracted lactating samples. More significantly, in lactating samples, the top 6 to 8 most abundant genes, corresponding to highly expressed milk protein genes, represent more than 50% of all ESTs in contrast to no more than 10% in non-lactating samples. Thus, a handful of milk protein genes account for a large proportion of transcripts in lactating samples while the expression of these genes is less dominant in pregnant or un-stimulated gland. This confirms how the lactating mammary gland is indeed specialised in the production of an increasingly massive amount of milk protein during lactation..

### Differential gene expression during the lactation cycle estimated from stage specific library sequencing data

In order to identify differentially expressed genes the data were normalized by pooling libraries from identical stages and used to perform a number of statistical tests using library sequence frequencies for a total subset of 971 informative super-clusters with at least one EST in any of the lactation phase specific libraries. The subtracted libraries at day 130 were kept in a separate pool. Tests were conducted using the IDEG6 online tool with a p-value > 0.001 applying the Bonferroni correction [27]. The analysis of library sequencing data resulted in the identification of a relatively small subset of 25 transcripts with significant differential expression across the lactation cycle of the tammar (highlighted in Additional file 1). This low number may reflect the limitation of shallow library sequencing and therefore nay not directly represent the number of genes that might be differentially expressed in the mammary gland during lactation. Indeed most of the transcripts identified represent high abundance transcripts that can be grouped in 5 sub-sets based on their expression profile: 1) ubiquitous milk protein transcript with higher expression in lactating than pregnant mammary glands, 2) genes with inverse expression profile, high during pregnancy, lower in lactation, 3) transcripts with high expression in mid lactation, only identified using the subtracted libraries at day 130 with improved coverage, 4) late lactation specific transcripts, 5) highly expressed transcript isolated from the unsucked gland. The first set consists of milk proteins more highly expressed in lactating than pregnant mammary glands: beta-casein, beta-lactoglobulin, kappacasein, alpha-lactalbumin, trichosurin (a marsupial specific milk protein originally characterised in the possum *Trichosurus vulpecula *[21]), ferritin heavy chain and two new potential tammar milk proteins PTMP-1 and PTMP-2-GlyCAM. Surprisingly, PTMP-1 seems to be highly specific to the tammar since no similarities could be found in the opossum or other available genome sequences. Significantly, this new predicted 132 amino acid open reading frame carries a high scoring secretory signal but BLAST or psi-BLAST [22] searches did not retrieve significant public database hit. Peptide analysis did not identify any known conserved motif beside the signal peptide. However, the partial genome sequence of the tammar wallaby contains sequence reads with high similarity to this transcript confirming the true tammar origin of this new candidate milk protein. In spite of this, a similar gene is not detected in the genome sequence of the American marsupial opossum and further experiments will be needed to assess the significance of this sequence for marsupial lactation and its distribution in the marsupial lineage. The other new putative tammar protein PTMP-2-GlyCAM presents no clear similarity to known proteins except for a predicted Glycosylation-dependent cell adhesion motif (Pfam:PF05242.1).

Interestingly, this glycosylation dependent cell adhesion domain is found in only two characterised proteins with clear relation to milk: lactophorin precursor proteose peptone component 3 (PP3) [28] and glycosylation-dependent cell adhesion molecule 1 (GlyCAM-1) [29]. GlyCAM-1 is expressed in the mammary gland of pregnant and lactating mice as well as human milk cells while PP3 has been identified in bovine milk [30]. In addition, expression of GlyCAM-1 in mice was positively regulated by prolactin [31] and further analysis has confirmed that bovine PP3 and mouse GLYCAM-1 are homologous [32]. The second group contains only two genes with inverse expression profile, namely, high during pregnancy, lower in lactation: ELP previously described as an early phase specific whey protein in the tammar [33], and cytochrome oxidase subunit I. The third set, which is only identified using the subtracted library at day 130, contains transcripts with high expression in mid-late lactation including the major WAP, known to be specifically expressed between day 130 and 240 of lactation [34], polymeric immunoglobulin receptor, cystatin C and transferrin. The fourth group represents late lactation specific transcripts containing the extensively characterised marsupial specific late lactation proteins LLP-A and LLP-B [11,17]. The fifth group contains highly expressed transcripts isolated from the unsucked gland: galactosidase-beta-1, three non coding transcripts PTNC-0, PTNC-1, and PTNC-2 as well as, specific to one of the libraries 19, 12 S ribosomal RNA and two repetitive sequences. Remarkably, three novel non-coding RNA-like sequences were identified amongst the 10 most abundant transcripts. PTNC-0 is a novel major transcript (6% in each libraries). PTNC-1 corresponds to a region of the genome that is highly conserved in all mammals including humans and opossum and overlaps the non-coding metastasis-associated lung adenocarcinoma transcript 1 (MALAT-1). Expression of MALAT-1 has been associated with Endometrial stromal sarcoma [35]. PTNC-2 is apparently of mitochondrial origin. In all, these results contain all the presently know tammar milk proteins and are in agreement with published phase-specific expression measurements. In addition they exhibit two new tammar milk protein candidates PTMP-1 and PTMP-2, suggest putative high mid-late lactation expression of a number of immune genes and indicate high expression of non coding transcripts in the 4-days unsucked gland..

### Massively parallel sequencing of lactating mammary gland at day 151 and 240 of lactation

To compensate for the limitation of sequencing cDNA libraries from lactating mammary tissue with a high proportion of milk protein transcripts, a more comprehensive massive sequencing approach was used. Massively parallel signature sequencing data [36] were obtained from the commercial provider LYNX Bioscience using polyA+ mRNA samples from the lactating mammary gland of individual tammar wallabies at day 151 and 240 of lactation. This represented 1,869,010 and 1,822,160 17-base pair sequence signature occurrences respectively from a total of 3,157 distinct signature sequences including 392 signatures common to both samples.

**Figure 4:**
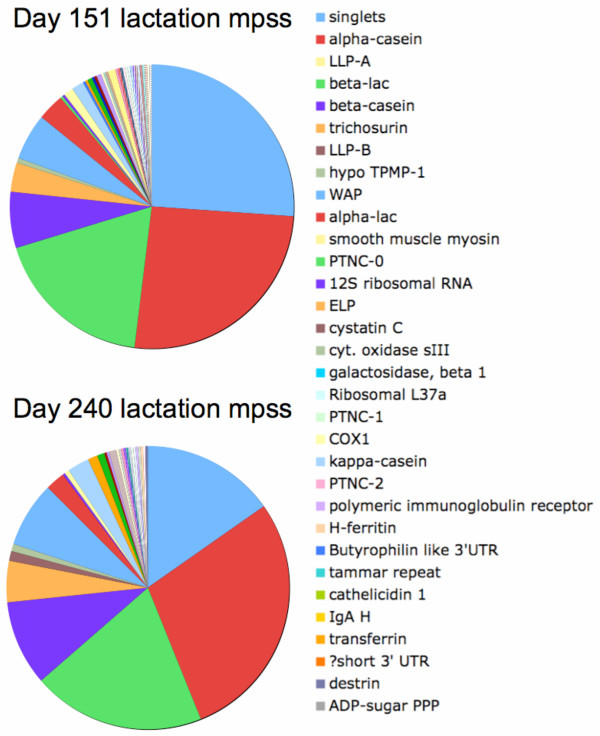
**Proportional composition of MPSS data from day 151 and day 240 of lactation**. Relative abundance of transcript was calculated by estimating the frequency of each transcript from the number of MPSS tags mapped to contig sequences (normalised to parts per 10000).

Simple clustering of signature sequences was used to compensate for sequencing errors, especially in high abundance transcripts leading to 2,521 independent signature clusters. Searching for the sequence signatures in the complete EST sequence database identified a total of 564 independent signature clusters (25% of all independent signatures) representing 72% (1,354,268 counts at day 151) and 80% (1,456,392 at day 240) of all MPSS counts showing that although only about a quarter of all signature clusters can be identified in the sequence assembly, they represent a large proportion of the transcript population. This process resulted in a comprehensive annotation of the tammar mammary gene catalogue with expression estimates from MPSS data. The resulting proportional compositions of transcript levels at day 151 and 240 of lactation are presented in Figure 4, showing a profile globally similar to lactating profiles obtained from EST sequencing in Figure 3. The integrated normalized quantification from EST and MPSS sequencing data for the most abundant genes identified are shown in Additional file 1. A contrast analysis between day 151 and 240 lactation MPSS data was performed using 335 EST clusters with at least one signature occurrence in any sample. This resulted in 225 transcripts with significant (p < 0.001) differences in all tests available and 25 additional transcripts passing at least one of the proposed statistical tests. The use of a much more stringent cut off (p < 0.00001) still identified 219 to 229 transcripts. In total this represents about 60% of the clusters with MPSS signature. These results suggest that significant changes in transcript levels occur in the lactating tissue between day 151 and 240 of lactation corresponding to the phase at which milk protein production is strongly increased. This contrast between shallow and deep sequencing results illustrates the effect of sampling on statistical resolution and is mainly influenced by the very small day-260 library sequencing sample. There are also limitations in the MPSS approach.

The use of a specific restriction site to target signature sequencing (dpnII) excludes transcripts missing the restriction sequence. For example LLP-A, a major late lactation milk protein, is not detected in this experiment. This might have a significant effect on expression level estimations when a high abundance gene is not detectable and missing data for such genes alone may bias the results of statistical tests.

### Prediction of secretory proteins and expression of the secretome

Open reading frames were predicted using ESTSCAN2 and submitted to the identification of signal peptides by IPSORT software [37] or SignalP web server [38].

This includes 145 IPSORT, 164 SignalP Neural network and 114 SignalP HMM predictions. Supercontig clustering resulted in a total of 213 supercontigs predicted by either IPSORT or SignalP to encode proteins with a signal peptide. This represented 140 IPSORT, 156 SignalP Neural network and 108 SignalP HMM predictions with 73 predictions common to all methods. Because different methods failed to identify all the previously described milk proteins, the larger union set of 213 predictions obtained by any method were retained for further analysis of the secretome in the mammary gland.

**Figure 5:**
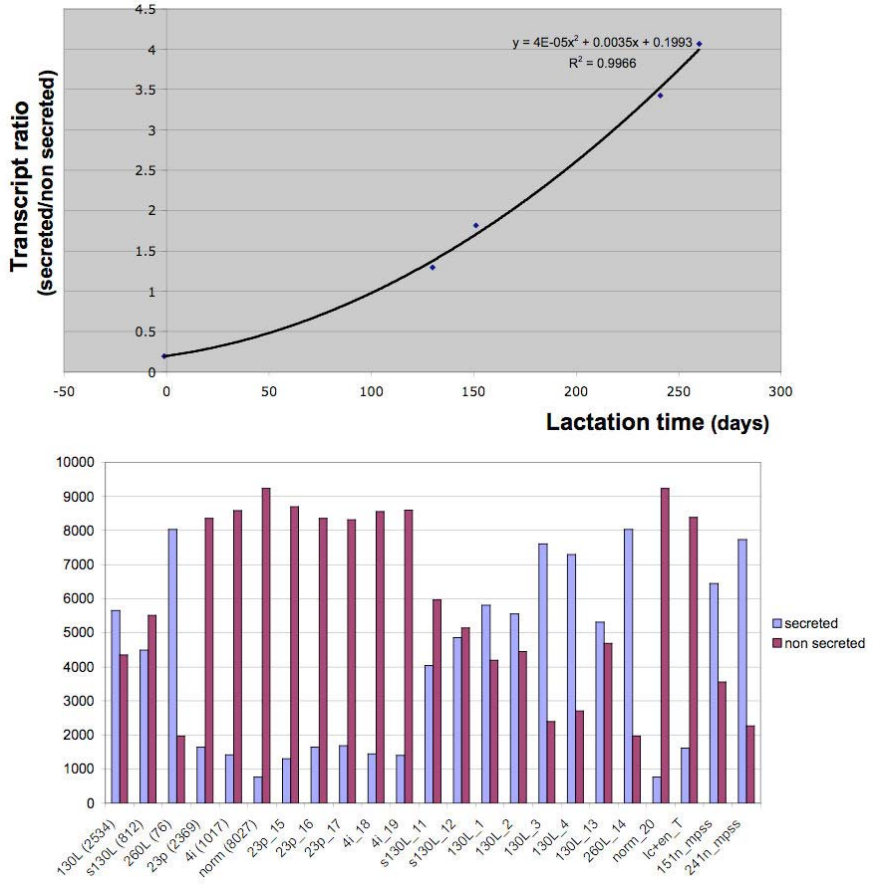
**Differential expression of the secretome during lactation**. Top: the ratio of transcripts encoding predicted secretory versus non-secretory proteins is shown in function of time (days) after combining EST sequencing (day 23 pregnant, day 130 and 260 of lactation) and MPSS data (day 151 and day 240). The proportion of secretory gene products increases consistently during lactation fitting a second degree polynomial (black line: R > 0.995). Bottom: proportion of predicted secreted protein gene transcript (blue) and other (red) (normalized to 10000) for the complete data set including library sequencing and MPSS. From left to right: pooled data (day-130 lactation, day-130 lactation subtracted, day-260 lactation, day-23 pregnant, day4 lactation unsucked, normalized library), individual stage specific libraries; day-23 pregnant (libraries 15, 16 and 17) day-4 lactation unsucked gland (libraries 18 and 19), day-130 lactation subtracted (libraries 11 and 12), day-130 lactation (libraries 1,2,3,4 and 13), day-260 lactation (libraries 11 and 12), normalised library, day-151 lactation mpss, day-240 lactation mpss.

In order to address global expression levels of secreted proteins in the mammary gland during lactation, library sequencing and MPSS experiments were combined to estimate the proportion of mammary transcripts encoding secreted proteins. This is shown in Figure 5, in which a steady increase in expression of predicted secreted protein transcripts during lactation can be seen. This increase in secreted protein expression correlates gene expression levels with growth of the mammary gland, growth of the young, increased milk production and milk protein synthesis, which all steadily increase during the lactation cycle [18].

### Transcription profile in late pregnancy

In the pregnant mammary gland, milk protein gene expression (beta-lactoglobulin, alpha-beta-kappa-caseins, alpha-lactalbumin, PTMP-1, PTMP-2-GlyCAM, trichosurin) estimated from the pool of day 23 pregnant samples represent only 6% of transcripts compared to an increasingly massive 45–75% in lactating samples. In addition, the most abundant transcript identified in this stage corresponds to ELP. However, this transcript is estimated at 2.1%, also a sharp contrast with much higher 15–20% levels for alpha-casein or beta-lactoglobulin during lactation. Cathelicidin 1, an immune related protein recently identified in the milk of mice and humans [35], is found at levels of 1%. Beside milk protein genes, a number of cytochrome oxidase subunits are consistently detected at relatively high levels (sI 2%, sII (1%), sIII 1%).

Below 1% abundance, translational machinery (EF1A, ribosomal proteins), energy metabolism (cytochrome b, cytochrome oxidase, NADH dehydrogenase s1, s2, s6) and immune system components dominate. These results paint a profile of the late pregnant mammary gland with a dominance of transcriptional machinery, energy metabolism and immune related components, except for a much lower level of milk protein transcripts reflecting how the late pregnant gland is prepared and primed for the rapid synthesis of milk after parturition.

### Transcription profile in mid lactation

Milk protein genes largely predominate in the day-130 expression profile: alpha, beta and kappa-caseins, beta-lactoglobulin and trichosurin accounting together for 45% of total transcripts. Other highly expressed transcript represent the new potential milk protein identified above PTMP-1 (2.7%), cytochrome oxidase (2.5%), cathelicidin 1 (0.6%), a novel putative non coding RNA PTNC-0 (0.5%), early lactation protein (ELP, 0.4%). At lower frequencies (> 0.1%), the profile is mainly dominated by: ribomosomal proteins, immunological proteins (IgA, MHC classII, kappa immunoglobin, butyrophilin) and, components of energy metabolism (NADH dehydrogenase, ATP synthetase, cytochrome oxidases). In addition, two milk proteins typically expressed at high level in late lactation, WAP and LLP-A, are detected at low levels (0.1%).

MPSS data from a day 151 lactating gland shows a similar trend with the major milk protein represented by up to 63% of MPSS signatures. However this also includes higher levels of WAP (5%) consistent with the increased expression of WAP between day 120 to 200 [34]. Expression of cytochrome C oxidase subunit 1 (1.1%) and a number of hypothetical proteins are also identified with abundance above 0.5%: a putative protein PTMP-3 with high similarity to a hypothetical protein (MGC52838) with homologues of unknown function in many species (0.7%), the hypothetical new putative tammar specific milk proteins PTMP-1 and PTMP-2-GlyCAM discussed above and, a short unassigned 3' EST (~0.5% each). PTMP-3 is found only in the day 151 MPSS sample and the normalized library. TPMP-2-GlyCAM is found in the day 151 MPSS as well as day 130 and pregnant libraries. PTMP-2-GlyCAM may be a new milk protein expressed specifically during early-mid lactation in the tammar and PTMP-3 may play a role in mid lactation. The remaining MPSS profile is again largely dominated by transcription machinery and energy metabolism.

### Transcription profile in late lactation

Interestingly, despite the relatively small number of sequence samples obtained, the day-260 library reveals some unique features of marsupial lactation. Out of a total of 76 sequences, 62 (83%) are included in 10 distinct contigs. Three singletons contain chimeric DNA with a region highly similar to contigs (LLP-A, LLP-B and 1 hypothetical protein), 3 are simple sequences probably resulting from poor sequencing while the remaining 8 unambiguously represent relatively short 3' cDNA regions. The most abundant transcript is late lactation protein A. Expression of this marsupial specific protein with similarity to lipocalins has been shown to be highest in late lactation [17]. LLP-A represents 22% of sequences in the day 260 library. The data also show that small but measurable amount of LLP-A sequences can be identified in pregnant and day-130 lactation samples at low levels varying between 0.1 to 0.7 %. In contrast, LLP-B is also present a high expression level at day 260 (11%) but, is not detected in other stages. Other milk proteins are beta-lactoglobulin (13%), alpha and beta-caseins (8 and 7.5%), alpha-lactalbumin (4%), kappa-casein (2.6%) and trichosurin (1.3%). Expression of all these genes can be detected throughout the lactation cycle. However, expression levels are consistently much higher in lactating than pregnant samples. The other two genes consist of cytochrome C oxidase III which represent 2.6% at day 260 or 130 of lactation and 1.1 % in pregnant samples and, the new putative tammar milk protein sequence (PTMP-1) representing 8% of transcript and also expressed at seemingly increasing levels during lactation (0.5% in pregnancy and 2.5% at day 130).

In parallel with sequencing the day-260 lactation cDNA library, an MPSS experiment was conducted on a slightly earlier sample from a day-240 lactating gland. There are also limitations in this approach such as the failure to detect LLP-A, a major late lactation milk protein due to absence of a dpnII restriction site. LLP-B is identified at about 1%. This is consistent with the description of a sharp increase in LLP-B expression after day 200 [11]. Other major milk proteins are found at high levels mostly correlated with the day-260 results. Except for alpha-casein which represent 8% of transcripts in the day-260 library and 18% in day-240 MPSS and trichosurin (1.3 and 4.8% respectively), quantitative estimates for the major milk proteins including beta-lactoglobulin, beta and kappa-caseins, alpha-lactalbumin and transferrin show more comparable normalised levels (within 30% of each other) in day-240 and day-260 glands from deep MPSS and shallow library sampling. Due to the limited cDNA library sampling at day 260 it is difficult to extract further information from these data but, MPSS data reveals two other genes that are expressed at high level including: the major whey acidic protein WAP (7.5%) known to be specifically expressed in phase 2b of lactation and, a 3' UTR (PTMP-4/Contig1786) with similarity to the solute carrier family 20 phosphate transporter SCL20A2 (1%), a protein that is likely expressed on the basolateral membranes of polarized epithelial cells, and is likely involved in cellular phosphate homeostasis [39].

### Transcription profile in unsucked gland

In cDNA libraries derived from un-sucked glands 4 days after parturition, milk protein transcripts represent 8 to 9 % of all transcripts, with predominance of betalactoglobulin (2.7%), PTMP-1 (2%), alpha- lactalbumin and alpha-casein (1.5% each), ELP and cathelicidin (0.5% each). Important differences are also observed in the two independent library samples. In general milk protein transcripts are less abundant in library 19 and this library is enriched in repetitive elements and ribosomal RNA as well as immune response markers. These observations may reflect stress, a pathological condition or a more advance stage of this sample toward regression of the gland.

High expression of beta-galactosidase (3%) is specific to library 18. This observation suggests carbohydrate degradation may play a role in un-sucked gland, possibly to reduce the contribution of free lactose in regulating milk volume by osmolarity. This aspect is however controversial as tammar and more generally marsupial milk is poor in lactose, the canonical milk osmol in many mammals [40]. It is noteworthy that the level of free lactose in wallaby milk is higher during the first 4 days of lactation when lactose is the main milk carbohydrate. Subsequently lactose declines to low levels when complex oligosaccharides first appear and increase gradually thereafter [41].

This critical 4-day period may account in part for the difference between the two tissue samples taken around day 4. Currently, the data do not include equivalent lactating mammary gland at 4 days to confirm a specific role of beta-galactosidase in un-stimulated tissue. Nevertheless high expression of beta-galactosidase points to a possible role of sugar degradation in un-sucked glands.

Remarkably, 3 sequences related to non-coding RNA, including PTNC-0 (6%), PTNC-1 and PTNC-2 (2% each), are identified at high level in both libraries. Transcripts for ribosomal proteins, immune related and energetic metabolism components were below 1% but dominated this background with differences between the two libraries as discussed above.

These results show slightly higher or comparable levels of milk protein gene transcripts in un-sucked compared to late pregnant glands, except for ELP and cathelicidin 1 and suggest surprisingly high levels of three enigmatic non-coding RNA. In addition it is suggested beta-galactosidase may play a role in the inhibition of lactation in un-sucked glands.

## Conclusion

cDNA sequencing of the lactating tammar wallaby mammary gland mRNA has permitted the identification of a large number of tammar genes expressed during lactation providing a catalogue representing about 25% of the tammar genome. This currently is the largest cDNA resource from a marsupial organism as library sequencing has been described in only one other species: the Australian northern brown bandicoot [42]. About 60% of cDNAs possess significant similarity with genes from other mammalian species and a larger proportion (95%) could be retrieved from the partial tammar wallaby genome sequence allowing for further characterisation of full-length sequences and associated genes. Sequence analysis suggested that about 10% of mammary transcripts might be marsupial specific and 15% mammal specific. While many milk proteins are conserved between mammals, other major transcripts identified in this study provide extensive illustration for marsupial specific milk protein genes including ELP, LLP-A, LLP-B, trichosurin and the new protein PTMP-2. In addition, an ortholog of the new potential milk protein PTMP-1 cannot be recovered from the American marsupial opossum genome sequence representing, together with a number of specific repetitive elements, other examples of lineage specific transcripts. At the other extreme, PTNC-1 a novel non-coding RNA is derived from a region of the genome that is ultra-conserved in mammals suggesting an important functional role.

Shallow sequencing of lactation phase specific cDNA libraries has allowed the estimation of phase-specific gene expression of major mammary transcripts, the characterization of a number of new putative milk proteins including genes apparently unique to the tammar, the identification of phase specific markers and, suggested a role for non-coding RNA and carbohydrate degradation during initialisation of lactation. Comparison of expression profiles estimated at different times of the lactation cycle from a combination of deep MPSS and shallow cDNA digital sequencing experiments revealed that, in general, actively lactating glands are expressing a limited number of common or phase specific milk protein genes at high and increasing levels accounting for up to 60% of all transcripts during late lactation.

In addition, a large proportion of genes represent translational machinery components, immune related product or genes involved in energy production. Not surprisingly, these observations show that the lactating mammary gland is an organ highly specialised in the synthesis of milk. In addition, the recovery of all previously characterised phase-specific milk proteins and the identification of a new potential phase-specific milk protein genes demonstrate some of the unique aspects of marsupial lactation with significant changes in milk composition during an elongated lactation period. Our observations from the mammary tissue of late pregnant animals have shown how the late pregnant mammary gland is primed for the rapid commencement of milk production after parturition. Similarly, the large increase in protein content of tammar milk during mid to late lactation is accompanied by an increase in the mammary gland expression of genes encoding secretory proteins. This increased gene expression correlates with growth of the mammary gland, growth of the young, milk production and milk protein synthesis, which all steadily increase during the lactation cycle [18,43]. This global change of gene expression in the glandular tissue may reflect a combination of changes in cellular gene expression and cell type populations within the tissue. As the mammary gland size steadily increases during the lactation cycle, the progressive replacement of the stroma by alveolar tissue during the course of pregnancy and lactation, and a marked increase of alveolar size during late lactation have been described [43]. It is not possible to deduce from current data if the increase in relative abundance of milk protein transcripts corresponds to an increase of milk protein gene expression in mammary epithelial cells (lactogenesis) only or, an increase in the number and proportion of secretory epithelial cells in the mammary gland during lactation (mammogenesis). Our observations result most likely from a combination of these processes.

The combination of cDNA and signature digital sequencing methodologies has highlighted some of the caveat and limitations of sequencing approaches for the study of gene expression in the highly specialised mammary gland. In this tissue, a large dominance of milk protein transcripts makes cDNA sequencing a less cost-effective method for gene discovery and produces a significant amount of noise in signature sequencing due to the accumulation of sequencing errors in dominant signatures.

These effects limit the depth and resolution of this approach for the analysis of the larger number of expressed genes. One advantage of digital sequencing for the estimation of gene expression over differential gene expression estimation by microarray is that it provides a more precise quantitative estimation of relative mRNA levels for the most highly expressed genes. However, our data also include a larger number of ESTs obtained from a normalised cDNA library using a combined set of samples covering the full duration of the lactation cycle. The expression of many of these genes represented by a single or few EST sequences cannot be discussed here but this unique EST resource will allow the development of a tammar mammary cDNA microarray to assess in more detail the changes in expression of a larger number of genes during lactation and also allow investigation of the molecular, hormonal and cellular mechanisms involved in the adaptation of lactation in the tammar wallaby and other marsupial species.

## Methods

### Animal husbandry

Tammar wallabies were housed on site at The Department of Primary Industries, Attwood, in open grassy yards. The grass diet was supplemented with ad libitum access to supplementary feed (Wallaby pellets, Ridley AgriProducts Pty Ltd) and water. All experimental procedures were approved by the DPI Attwood Animal Ethics Committee (#2179).

### RNA preparation

All RNA samples were obtained from different animals. Total RNA was prepared using TRIZOL Reagent (Invitrogen). This involved homogenisation of frozen tissue in a mono-phasic solution of phenol and guanidine isothiocyanate followed by extraction of the aqueous phase with chloroform and precipitation of the RNA using ethanol. Precipitated RNA was then resuspended in RNase free water (Invitrogen).

Where indicated, mRNA was purified from total RNA using oligo dT attached to magnetic beads (PolyATract, Promega).

### cDNA library construction and DNA sequencing

The cDNA sequences were produced from lactation stage specific cDNA cloning of mRNA obtained from day 23 pregnant, day 130, day 260 of lactation or 4-day unsucked gland. First strand cDNA was prepared from total RNA (1 ug) or mRNA (500 ng) using the SMART II oligonucleotide and MMLV reverse transcriptase as recommended by the manufacturer (Promega) except for library 15, for which a thermostable reverse transcriptase (Thermoscript, Invitrogen) was used. PCR was used to make second strand cDNA according to the SMART system instructions (Promega). The first strand cDNA was then ligated with pGEMTeasy (Promega) using the recommended ligation reaction conditions and reagents. In some cases, the second strand cDNA was treated further prior to ligation when size selection of cDNA molecules was achieved by two methods. For libraries 5–10, the cDNA was fractionated by agarose gel electrophoresis and fragments of different sizes were purified using silicon beads (Qiaex beads, Qiagen). For libraries 16–19, small cDNA fragments were removed by Sephadex column chromatography using a CHROMA SPIN-1000 column (Promega). Electroporation was used to transform E. coli DH5a with ligated material and recombinant bacteria were selected for ampicillin resistance.

Subtracted libraries were also produced; for two libraries (11 and 12), cDNA for alpha-lactalbumin, beta-lactoglobulin, alpha-casein, beta-casein and kappa-casein was removed from the cDNA pool following the manufacturers instructions for the SMART subtraction library (Promega) except that the subtracted material used was double stranded cDNA corresponding to the five genes listed above.

In addition, a commercial normalised library (Life Technologies, Rockville, MD, USA) was also obtained using a propriety subtraction based approach to minimise the abundance of cDNAs representing milk protein genes. Total RNA isolated from tammar wallaby mammary gland during lactation (days 55, 87, 130, 180, 220, 260) and involution (5 days after removal of pouch young at 45d lactation) were pooled and used for normalised library construction. Subsequent cDNA molecules were directionally cloned into pMCVSport6-ccdB vector using NotI (3') and EcoRV (5') sites. Quality control of the resulting library revealed a titre of 1.2 × 10e5 cfu/ml with a mean insert size of 1.2 kb. Southern hybridisation for abundance of alpha-casein cDNA molecules revealed a reduction of 37 fold (7.8% in the non-normalised library, 0.2% in the normalised library).

Insert DNA was characterised by sequence determination (ABI 3700 DNA sequencer) and subsequent comparison to the public database (BLASTn, BLASTx).

Directional sequencing from the 5' end of the cDNA insert was achieved through use of the SP6 primer (Promega).

### Sequence assembly and annotation

EST Sequences were obtained using Phrap base calling software and assembled using Phred and Cap3 software. In general, the Cap3 assembly resulted in tighter clustering and was therefore retained for further analysis. Sequence mapping and annotation were conducted using the EST-PAC platform incorporating ncbi BLAST suite, Estscan2 and HMMer software in a web oriented database system [44]. Estscan2 translation were used for the prediction of signal peptide by IPSORT [27] and SignalP [38]. For the Multi-species comparison of mammary transcript sequences, the blast algorithm was used to identify top hits obtained using the tammar contig sequences against Unigene sequence data sets (dog, rat, mouse, cow) or cDNA sequences retrieved from the Ensembl annotation of a number of species (tenrec, man, chicken, armadillo, zebrafish, opossum and platypus). Percentage sequence similarity of cDNA top hit BLAST alignments between the tammar and other species were retrieved from the BLAST output stored in EST-PAC using cut off e value > 1e-8 and selecting alignment longer than 100 nucleotides.

### MPSS

MPSS was commercially provided by LYNX Bioscience using mRNA obtained from lactating mammary gland of tammar wallabies at day 151 or 240 post-partum using the restriction enzyme dpnII prior to signature sequencing [36]. Mpss gene mapping and quantitative analysis of stage specific library sequencing was done using custom scripts written in PHP language. A total of 1,869,010 and 1,822,160 17-base pair sequence signature occurrences respectively were obtained from a total of 3,157 distinct signature sequences including 392 signatures common to both samples among 2,350 from the day-151 sample and 1199 signature from day-240. Interestingly, the common set of 392 signatures represented a large proportion of the data: 79% (1,483,838) for day-151 and 90% (1,657,794) for day-240. To compensate for sequencing errors, especially in high abundance transcripts, simple clustering of signature sequences was conducted using an edit distance between sequence signatures compatible with sequencing errors (allowing for 1 error or mismatch past the required restriction site) and resulted in 287 clusters containing 923 signatures and 2,234 unique sequence signatures. This included 118 clusters containing signatures common to both samples representing a large majority of counts: 76% (1,436,813) for day 151 and 89% (1,630,347) for day 240 samples. Signatures were subsequently mapped to either contig or EST sequences using a perfect match search for all signature variations within a signature cluster. This resulted in the identification of 86 out of 287 signatures clusters in contig sequences, representing 64% and 79% of all MPSS data in day 151 and 240 samples. In addition, searching for all sequence signatures in the complete EST sequence database identified a total of 564 independent signatures (25% of all independent signatures) representing 72% (1,354,268 counts at day 151) and 80% (1,456,392 at day 240) of all MPSS counts.

Using imperfect matching did not improve the annotation significantly. Finally, the data were manually inspected in order to resolve conflicts when a signature produced a match with two different genes. In most cases this could be resolved by investigating the proximity of the matching position to the 3' poly-adenylated extremity of the transcript sequence and the presence of additional dpnII restriction cleavage site in this interval, excluding sequence with additional sites as primary hits.

Statistical analysis of sequence counts was done using the IDEG6 web interface [27].

## Authors' contributions

Kevin, Matthew and Christophe implemented the study. Jane conducted molecular cloning and sequencing. Christophe directed data analysis. Yvan was involved with automatic and Matthew with manual annotation. Christophe wrote the paper with assistance from Matthew and Kevin. All authors read and approved the final manuscript.

## Supplementary Material

Additional File 1 Normalized abundance of major mammary transcripts expressed as parts per 10000 (ordered by decreasing maximal abundance across the lactation cycle). Genes identified as differentially expressed are highlighted: ubiquitous milk protein group 1: blue, pregnancy group 2: green, mid-late lactation group 3: purple, late lactation group 4 ; red, unsucked gland group 5; orange. Click here for file
