# Supplementary material for: Lactation transcriptomics in the Australian marsupial, Macropus eugenii: transcript sequencing and quantification
Source: BMC Genomics. 2007 Nov 13;8:417. doi: 10.1186/1471-2164-8-417 (PMC2204018; doi:10.1186/1471-2164-8-417)
Supplement: Additional File 1 — Normalized abundance of major mammary transcripts expressed as parts per 10000 (ordered by decreasing maximal abundance across the lactation cycle). Genes identified as differentially expressed are highlighted: ubiquitous milk protein group 1: blue, pregnancy group 2: green, mid-late lactation group 3: purple, late lactation group 4 ; red, unsucked gland group 5; orange. [file 1471-2164-8-417-S1.doc]

Table 2: Normalized abundance of major mammary transcripts expressed as parts per 10000 (ordered by decreasing maximal abundance across the lactation cycle). Genes identified as differentially expressed are highlighted: ubiquitous milk protein group 1: blue, pregnancy group 2: green, mid-late lactation group 3: purple, late lactation group 4 ; red, unsucked gland group 5; orange.

| **ContigID** | **23p (2369)** | **4i (1017)** | **130L (2534)** | **s130L (812)** | **260L (76)** | **151 mpss** | **240 mpss** | **norm (8027)** | **max** | **Name** |
| --- | --- | --- | --- | --- | --- | --- | --- | --- | --- | --- |
| Others | 2596.0 | 4867.3 | 1641.7 | 270.9 | 1315.8 | 2615.4 | 1531.9 | 4458.7 | 4867.3 | singlets |
| Contig147 | 16.9 | 0.0 | 11.8 | 12.3 | 2236.8 | 0.0 | 0.0 | 2.5 | 2236.8 | LLP-A |
| SUPER5 | 126.6 | 137.7 | 2213.9 | 270.9 | 789.5 | 1580.8 | 1855.1 | 22.4 | 2213.9 | alpha-casein |
| SUPER4 | 177.3 | 275.3 | 1097.1 | 652.7 | 1315.8 | 1835.1 | 1968.9 | 11.2 | 1968.9 | beta-lac |
| Contig146 | 0.0 | 0.0 | 0.0 | 0.0 | 1184.2 | 1.1 | 98.4 | 1.2 | 1184.2 | LLP-B |
| SUPER11 | 21.1 | 9.8 | 611.7 | 640.4 | 657.9 | 618.9 | 980.6 | 12.5 | 980.6 | beta-casein |
| SUPER6 | 59.1 | 196.7 | 272.3 | 246.3 | 789.5 | 57.4 | 78.0 | 5.0 | 789.5 | hypo PTMP1 |
| Contig1 | 0.0 | 0.0 | 11.8 | 344.8 | 0.0 | 528.3 | 749.5 | 2.5 | 749.5 | WAP |
| Contig452 | 12.7 | 619.5 | 47.4 | 0.0 | 0.0 | 0.0 | 0.0 | 3.7 | 619.5 | PTNC-0 |
| Contig16 | 25.3 | 0.0 | 150.0 | 1182.3 | 131.6 | 341.1 | 474.7 | 8.7 | 474.7 | trichosurin |
| SUPER10 | 92.9 | 147.5 | 371.0 | 135.5 | 394.7 | 292.4 | 243.3 | 1.2 | 394.7 | alpha-lac |
| SUPER3 | 114.0 | 29.5 | 264.4 | 61.6 | 263.2 | 0.0 | 0.0 | 1.2 | 264.4 | cyt. oxidase sIII |
| SUPER2 | 4.2 | 19.7 | 146.0 | 36.9 | 263.2 | 116.3 | 234.7 | 0.0 | 263.2 | kappa-casein |
| Contig352 | 215.3 | 49.2 | 39.5 | 295.6 | 0.0 | 0.0 | 0.0 | 7.5 | 215.3 | ELP |
| Contig462 | 4.2 | 206.5 | 11.8 | 0.0 | 0.0 | 16.2 | 21.2 | 2.5 | 206.5 | PTNC-1 |
| Contig31 | 4.2 | 196.7 | 98.7 | 0.0 | 0.0 | 0.0 | 0.0 | 2.5 | 196.7 | PTNC-2 mt ribosomal RNA |
| Contig4 | 194.2 | 29.5 | 35.5 | 36.9 | 0.0 | 111.3 | 56.7 | 1.2 | 194.2 | COX1 |
| Contig30 | 21.1 | 78.7 | 185.5 | 381.8 | 0.0 | 47.0 | 0.0 | 1.2 | 185.5 | hypo PTMP2 (glyCAM domain) |
| SUPER8 | 67.5 | 19.7 | 165.7 | 2931.0 | 0.0 | 6.9 | 6.2 | 18.7 | 165.7 | H-ferritin |
| Contig457 | 0.0 | 147.5 | 0.0 | 0.0 | 0.0 | 0.0 | 0.0 | 0.0 | 147.5 | galactosidase, beta 1 |
| Contig57 | 16.9 | 19.7 | 15.8 | 12.3 | 131.6 | 0.0 | 0.0 | 0.0 | 131.6 | IgA H |
| Contig123 | 4.2 | 0.0 | 3.9 | 0.0 | 131.6 | 0.0 | 0.0 | 0.0 | 131.6 | ?short 3’ UTR EF1 |
| Contig234 | 8.4 | 0.0 | 0.0 | 0.0 | 131.6 | 0.0 | 0.0 | 2.5 | 131.6 | destrin |
| Contig1462 | 0.0 | 0.0 | 0.0 | 0.0 | 131.6 | 0.0 | 0.0 | 2.5 | 131.6 | ADP-sugar PPP |
| Contig32 | 4.2 | 9.8 | 3.9 | 86.2 | 131.6 | 29.8 | 102.0 | 0.0 | 131.6 | transferrin |
| SUPER1 | 114.0 | 59.0 | 59.2 | 61.6 | 0.0 |  |  | 14.9 | 114.0 | cathelicidin 1 |
| SUPER34 | 101.3 | 29.5 | 35.5 | 98.5 | 0.0 | 0.0 | 0.6 | 0.0 | 101.3 | cytochrome c oxidase sII |
| Contig1786 | 4.2 | 0.0 | 0.0 | 0.0 | 0.0 | 1.1 | 96.8 | 2.5 | 96.8 | hypo PTMP4 SLC20A1 |
| SUPER38 | 0.0 | 88.5 | 0.0 | 0.0 | 0.0 |  |  | 0.0 | 88.5 | tammar repeat |
| Contig126 | 80.2 | 0.0 | 23.7 | 0.0 | 0.0 | 0.0 | 0.0 | 5.0 | 80.2 | ribosomal S3 |
| Contig359 | 71.8 | 78.7 | 39.5 | 12.3 | 0.0 | 0.0 | 0.0 | 2.5 | 78.7 | ?short 3’ possum Imune related est |
| SUPER12 | 25.3 | 0.0 | 23.7 | 0.0 | 0.0 | 57.4 | 78.0 | 0.0 | 78.0 | ?short 3’ B-lac? |
| Contig1210 | 0.0 | 0.0 | 0.0 | 0.0 | 0.0 | 73.5 | 0.0 | 8.7 | 73.5 | hypo PTMP3 |
| Contig308 | 67.5 | 9.8 | 3.9 | 24.6 | 0.0 | 15.7 | 5.3 | 1.2 | 67.5 | EF-1-alpha-1 |
| Contig172 | 67.5 | 9.8 | 23.7 | 12.3 | 0.0 | 0.0 | 0.0 | 6.2 | 67.5 | Ribosomal L37a |
| Contig293 | 59.1 | 0.0 | 11.8 | 12.3 | 0.0 | 4.6 | 0.5 | 2.5 | 59.1 | actin type 5 |
| SUPER37 | 21.1 | 59.0 | 7.9 | 12.3 | 0.0 | 32.1 | 27.8 | 5.0 | 59.0 | bone marrow antigene? |
| Contig473 | 0.0 | 59.0 | 0.0 | 0.0 | 0.0 | 0.0 | 0.0 | 0.0 | 59.0 | tammar repeat |
| Contig150 | 54.9 | 19.7 | 23.7 | 0.0 | 0.0 | 0.0 | 0.0 | 1.2 | 54.9 | ?bad sequence |
| Contig299 | 50.7 | 19.7 | 7.9 | 61.6 | 0.0 | 0.0 | 0.0 | 2.5 | 50.7 | immunoglobulin kappa light |
| Contig93 | 50.7 | 0.0 | 35.5 | 0.0 | 0.0 | 13.3 | 0.5 | 0.0 | 50.7 | ATPase s6 |
| Contig323 | 50.7 | 19.7 | 7.9 | 0.0 | 0.0 | 2.3 | 1.5 | 5.0 | 50.7 | Ribosomal L9 |
| Contig169 | 50.7 | 29.5 | 11.8 | 0.0 | 0.0 | 0.0 | 0.0 | 0.0 | 50.7 | S15 |
| Contig318 | 50.7 | 0.0 | 0.0 | 12.3 | 0.0 | 0.0 | 0.0 | 0.0 | 50.7 | cytochrome b |
| Contig495 | 4.2 | 49.2 | 3.9 | 0.0 | 0.0 | 0.0 | 0.0 | 2.5 | 49.2 | similar to putative RNA binding protein 1 |
| Contig122 | 46.4 | 0.0 | 7.9 | 49.3 | 0.0 | 0.5 | 0.5 | 6.2 | 46.4 | cyclophilin |
| Contig5 | 46.4 | 9.8 | 23.7 | 0.0 | 0.0 | 0.0 | 0.0 | 3.7 | 46.4 | ?short 3’ trichosurin |
| Contig307 | 46.4 | 0.0 | 11.8 | 0.0 | 0.0 | 0.0 | 0.0 | 1.2 | 46.4 | ribosomal S27 |
| SUPER16 | 42.2 | 19.7 | 15.8 | 0.0 | 0.0 | 2.1 | 0.2 | 1.2 | 42.2 | ribosomal S20 |
| SUPER19 | 42.2 | 19.7 | 23.7 | 0.0 | 0.0 | 2.8 | 0.7 | 1.2 | 42.2 | S15a |
| Contig184 | 42.2 | 0.0 | 7.9 | 12.3 | 0.0 | 10.9 | 8.3 | 3.7 | 42.2 | S14 |
| Contig397 | 42.2 | 29.5 | 11.8 | 0.0 | 0.0 | 0.0 | 0.0 | 1.2 | 42.2 | ribosomal L23 |
| SUPER14 | 42.2 | 19.7 | 31.6 | 0.0 | 0.0 |  |  | 3.7 | 42.2 | S7 |
| SUPER29 | 42.2 | 0.0 | 3.9 | 0.0 | 0.0 |  |  | 0.0 | 42.2 | heparin-binding HBp15 |
| Contig101 | 8.4 | 9.8 | 39.5 | 0.0 | 0.0 | 0.0 | 0.0 | 1.2 | 39.5 | ?short 3’ trichosurin |
| SUPER13 | 12.7 | 39.3 | 35.5 | 86.2 | 0.0 | 32.8 | 31.2 | 0.0 | 39.3 | apolipoprotein CI |
| Contig159 | 38.0 | 39.3 | 19.7 | 24.6 | 0.0 | 2.5 | 0.0 | 0.0 | 39.3 | Ribonuclease H1 |
| SUPER44 | 12.7 | 39.3 | 15.8 | 0.0 | 0.0 | 0.3 | 0.0 | 14.9 | 39.3 | putative RT |
| Contig479 | 8.4 | 39.3 | 7.9 | 12.3 | 0.0 | 0.0 | 0.0 | 1.2 | 39.3 | Cytochrome Bc1 |
| Contig331 | 8.4 | 39.3 | 3.9 | 0.0 | 0.0 | 0.0 | 0.0 | 14.9 | 39.3 | similar to LINE-1 reverse transcriptase |
| Contig346 | 38.0 | 29.5 | 27.6 | 12.3 | 0.0 | 3.6 | 1.7 | 0.0 | 38.0 | NADH dehydrogenase subunit 1 |
| Contig496 | 38.0 | 19.7 | 27.6 | 36.9 | 0.0 | 1.5 | 1.2 | 1.2 | 38.0 | S12 |
| Contig320 | 38.0 | 0.0 | 0.0 | 0.0 | 0.0 | 4.1 | 3.6 | 1.2 | 38.0 | NADH dehydrogenase s2 |
| Contig171 | 38.0 | 0.0 | 0.0 | 0.0 | 0.0 | 4.6 | 4.3 | 0.0 | 38.0 | Hypothetical LOC615556 |
| SUPER40 | 38.0 | 19.7 | 0.0 | 0.0 | 0.0 |  |  | 14.9 | 38.0 | immunoglobulin lambda light |
| Contig239 | 29.5 | 29.5 | 23.7 | 61.6 | 0.0 | 0.0 | 0.0 | 1.2 | 29.5 | similar to thymosin-like 4 |
| Contig491 | 12.7 | 29.5 | 0.0 | 36.9 | 0.0 | 1.6 | 0.0 | 2.5 | 29.5 | clusterin |
| Contig249 | 12.7 | 9.8 | 27.6 | 61.6 | 0.0 | 10.7 | 6.5 | 0.0 | 27.6 | Butyrophilin like 3’UTR |
| Contig1967 | 4.2 | 9.8 | 23.7 | 258.6 | 0.0 | 0.0 | 0.0 | 19.9 | 23.7 | cystatin C |
| Contig1235 | 12.7 | 19.7 | 3.9 | 49.3 | 0.0 | 2.3 | 0.2 | 7.5 | 19.7 | L8 |
| Contig33 | 4.2 | 0.0 | 15.8 | 209.4 | 0.0 | 9.5 | 0.0 | 3.7 | 15.8 | polymeric immunoglobulin receptor |
| SUPER7 | 12.7 | 0.0 | 0.0 | 49.3 | 0.0 | 9.3 | 0.3 | 2.5 | 12.7 | ubiquitin C |
| Contig21 | 12.7 | 9.8 | 11.8 | 49.3 | 0.0 | 0.0 | 0.5 | 2.5 | 12.7 | S29 |
